# A large, short-armed, winged dromaeosaurid (Dinosauria: Theropoda) from the Early Cretaceous of China and its implications for feather evolution

**DOI:** 10.1038/srep11775

**Published:** 2015-07-16

**Authors:** Junchang Lü, Stephen L. Brusatte

**Affiliations:** 1 Institute of Geology, Chinese Academy of Geological Sciences, Beijing 100037, China; Key Lab of Stratigraphy and Paleontology, Ministry of Land and Resources of China, Beijing 100037, China; 2 School of GeoSciences, University of Edinburgh, Grant Institute, James Hutton Road, Edinburgh EH9 3FE, United Kingdom

## Abstract

The famous ‘feathered dinosaurs’ from the Early Cretaceous of Liaoning Province, northeastern China, include several dromaeosaurids, which are among the closest relatives of birds. Most of these are small-bodied taxa with long arms and broad wings comprised of vaned feathers, but a single specimen (the holotype of *Tianyuraptor*) belongs to a much larger individual with reduced forelimbs, which unfortunately lacks any preserved integument. We describe a new specimen of large-bodied, short-armed Liaoning dromaeosaurid, which we designate as a new genus and species, *Zhenyuanlong suni*. The integument is well preserved and provides the first evidence of feather morphologies and distribution in a short-armed (and probably non-volant) dromaeosaurid, indicating that these rare and aberrant taxa had large wings consisting of pennaceous feathers on the arms and long pennaceous feathers on the tail very similar to their smaller and longer-armed relatives, but potentially lacked vaned feathers on the legs. *Zhenyuanlong* adds yet more diversity to the Liaoning dromaeosaurid fauna, helps further reveal a distinct short-armed bauplan among dromaeosaurids, and illuminates previously-unrecognized homoplasy that complicates dromaeosaurid phylogeny and suggests that the Liaoning taxa may not have formed their own clade.

Over the past two decades, the famous ‘feathered dinosaurs’ from Liaoning Province and surrounding areas in China have become some of the most iconic fossils in palaeontology. The thousands of downy-covered specimens from this exceptionally preserved Early Cretaceous biota beautifully illustrate the evolutionary transition from carnivorous theropod dinosaurs to birds^1^,^2^,^3^,^4^. Some of the most intriguing of these specimens are the basal paravian taxa that closely bracket the dinosaur-bird transition, many of which have ‘modern’ pennaceous flight feathers comprising large wings on the arms and, in some cases, the legs^5^,^6^,^7^. Among these are several species of dromaeosaurids, members of the diverse clade of close avian relatives that includes the sickle-clawed *Velociraptor*^8^,^9^.

The Liaoning dromaeosaurids include five genera, some of which may include more than one species: *Changyuraptor*^10^, *Graciliraptor*^11^, *Microraptor*^5^,^12^, *Sinornithosaurus*^13^,^14^ and *Tianyuraptor*^15^. These are mostly small animals, not much larger than a housecat or a medium-sized dog, which had long forelimbs and extensive arrays of vaned feathers. The one exception is the only known specimen of *Tianyuraptor*, which belonged to a much larger animal that approached two meters in body length, and which possessed reduced forearms that are only about half the length of the hindlimbs^15^. Unfortunately, feathers were not preserved on this specimen, leaving open the question of what type of integument short-armed dromaeosaurids had and whether they possessed large wings with pennaceous feathers like their smaller Liaoning relatives. This is an especially pertinent issue because, although the flight capabilities of smaller longer-armed dromaeosaurids are still uncertain^7^, the larger short-armed taxa were almost certainly incapable of advanced volant activity^15^. We currently do not know what types of feathers such non-volant paravians possessed.

We here report a second specimen of large-bodied, short-armed Liaoning dromaeosaurid (Fig. 1). This animal is slightly smaller than the holotype of *Tianyuraptor*, but has a proportionally shorter forelimb and differs in numerous other features, supporting its identification as a new taxon. Most importantly, this specimen has a well-preserved integument and provides the first information on feather morphology and distribution in a large, short-armed dromaeosaurid. Like other Liaoning dromaeosaurids, this new taxon has broad wings on its arms comprised of multiple sets of pennaceous feathers and large pennaceous feathers on the tail, but unlike many basal paravian and avialan taxa appears to lack vaned feathers on the hindlimb. The new taxon also increases the diversity of Liaoning dromaeosaurids, gives further evidence that long-armed and short-armed dromaeosaurids may have coexisted in the Early Cretaceous, and reveals a large amount of homoplasy that complicates the phylogeny of dromaeosaurids and indicates that the Liaoning taxa may not form their own distinct clade.

## Results

### Systematic palaeontology

Dinosauria Owen 1842. Saurischia Seeley 1887. Theropoda Marsh 1881. Coelurosauria Huene 1914. Maniraptora Gauthier 1986. Dromaeosauridae Matthew and Brown 1922. *Zhenyuanlong suni* gen. et sp. nov.

### Etymology

“Long”, from the Chinese Pinyin, means dragon. The generic and specific names are in honor of Mr. Zhenyuan Sun, who secured the specimen for study.

### Holotype

A nearly complete skeleton with skull and lower jaws preserved (JPM-0008), curated at the Jinzhou Paleontlogical museum. It is likely a sub-adult, as neural arches and centra are not fused in some anterior dorsal vertebrae and the sacral vertebrae, and the anterior sacral vertebrae are not completely fused to each other. The individual is fairly mature, however, as the more posterior sacrals are fused to each other and the neural arches and centra of the cervical vertebra, caudal vertebrae, and some dorsal vertebrae are fused.

### Type Locality and Horizon

Sihedang of Jianchang County, Liaoning Province; Yixian Formation^16^.

### Diagnosis

Dromaeosaurid theropod with the following unique combination of characters, with autapomorphies among dromaeosaurids indicated with an asterisk and autapomorphies among Liaoning dromaeosaurids indicated with a double asterisk: extremely slender radius whose shaft is thinner than the shaft of manual phalanax I-1*; a shortened metacarpal II, which is shorter than the combined lengths of metacarpal I and manual phalanax I-1** (differing from *Changyuraptor*, *Graciliraptor*, *Microraptor*, *Sinornithosaurus*, and *Tianyuraptor*, in which metacarpal II is longer than metacarpal I + phalanx I-1); six sacral vertebrae** (*Microraptor*, *Sinornithosaurus*, and *Tianyuraptor* have five or fewer, although additional unfused sacrals may be present and unpreserved in the latter); a shortened forelimb that is approximately half the length of the hindlimb, with a humerus:femur ratio less of than 0.65, an ulna:femur ratio of less than 0.55, and a manus:femur ratio of less than 0.90 (these features are shared with only *Tianyuraptor* among Liaoning dromaeosaurids, although this may be due to allometry given their shared larger size than other Liaoning dromaeosaurids^17^, and additionally *Mahakala* among all dromaeosaurids); absence of a prominent lateral tubercle at the midpoint of the lateral surface of the pubic shaft (also absent in *Tianyuraptor* and non-Liaoning dromaeosaurids, but present in *Changyuraptor*, *Microraptor*, and *Sinornithosaurus*^9^,^10^,^18^); absence of a distally placed dorsal process along the posterior edge of the ischial shaft (also absent in *Tianyuraptor* and most non-Liaoning dromaeosaurids, but present in *Microraptor* and *Sinornithosaurus*).

*Zhenyuanlong* differs further from *Tianyuraptor* in possessing an antorbital fossa bordered ventrally by a sharp rim (as in *Sinornithosaurus*); a pointed posterior end of the iliac postacetabular process (as in *Sinornithosaurus*, but lacking the lobate brevis shelf that projects beyond the end of the postacetabular lamina in *Microraptor* and *Tianyuraptor*); and a pubis with an anteriorly convex shaft (as in *Changyuraptor*, *Microraptor*, and *Sinornithosaurus*, but not straight as in *Tianyuraptor*). *Zhenyuanlong* also lacks ossified uncinated processes, which are present in *Tianyuraptor* and *Microraptor*, But this is not a definitive diagnostic difference because some Liaoning theropods are known from multiple specimens, some of which preserve uncinate processes and others of which do not.

Limb ratios are also slightly different in *Zhenyuanlong* and *Tianyuraptor*: the forelimb is shorter relative to the hindlimb (ratio of 0.48 vs. 0.53) and the manus is shorter relative to the femur (ratio of 0.76 vs. 0.86) in *Zhenyuanlong*. However, given that only two specimens are being compared, these differences are of questionable diagnostic value, without more information on intraspecific variation and possibly variation caused by taphonomic distortion. We note that the difference forelimb:hindlimb ratio is within the range of intraspecific variation in two paravian taxa known from multiple specimens, *Microraptor*^17^ and *Archaeopteryx*^19^,^20^, and is therefore not likely to be a clearly distinguishing feature between *Tianyuraptor* and *Zhanyuanlong*.

*Zhenyuanlong* differs further from *Microraptor* in possessing a manual phalanx III-1 that is less than twice the length of III-2 (the *Microraptor* condition is also present in *Sinornithosaurus*) and an iliac preacetabular process with a pointed anterior end (as in *Tianyuraptor*, but unlike the strongly convex and lobate morphology of *Microraptor*). Furthermore, *Zhenyuanlong* lacks two autapomorphies of *Microraptor*: distal maxillary teeth with a pronounced constriction and very strongly recurved and slender pedal unguals with prominent flexor tubercles^9^,^12^.

*Zhenyuanlong* differs further from *Sinornithosaurus* in possessing a first premaxillary tooth considerably smaller than the second and third (not approximately equal in size as in *Sinornithosaurus* and *Changyuraptor*), more elongate middle-posterior caudal vertebral centra, which are three times the length or greater than the dorsal vertebrae (as is also the case in *Microraptor* and *Tianyuraptor*) and an iliac postacetabular process that is acuminate (as in *Microraptor* and *Tianyuraptor*), not squared off. *Zhenyuanlong* also lacks the heavily pitted antorbital fossa anterolateral to the antorbital fenestra that is autapomorphic for *Sinornithosaurus*^21^.

*Zhenyuanlong* differs further from *Changyuraptor* in possessing a second premaxillary tooth that is considerably larger than the third and fourth teeth (as in *Sinornithosaurus*); a transition point between the 7^th^ and 10^th^ caudal vertebrae (as in *Microraptor* and *Tianyuraptor*, not proximal to the 7^th^ caudal vertebra as in *Changyuraptor*); a small semilunate carpal that covers approximately half of the bases of metacarpals I and II (as in *Microraptor* and *Tianyuraptor*, not the larger carpal that covers the entire proximal ends of metacarpals I and II in *Changyuraptor*); a straight metacarpal III (as in *Graciliraptor* and *Sinornithosaurus*, not the bowed morphology of *Changyuraptor*); a metatarsal IV that is approximately the same width as metatarsals II and III (as in *Microraptor* and *Sinornithosaurus*, unlike the proportionally broader metatarsal IV of *Changyuraptor*); and a metatarsal II that extends distally to the same level as metatarsal IV (as in *Sinornithosaurus*, but unlike the further distally extending metatarsal IV of *Microraptor* and *Changyuraptor*).

*Zhenyuanlong* differs further from *Graciliraptor* in that it lacks the extremely elongate and slender tibiotarsus that is autapomorphic of the latter taxon^9^,^11^.

### Description

**Figure 1:**
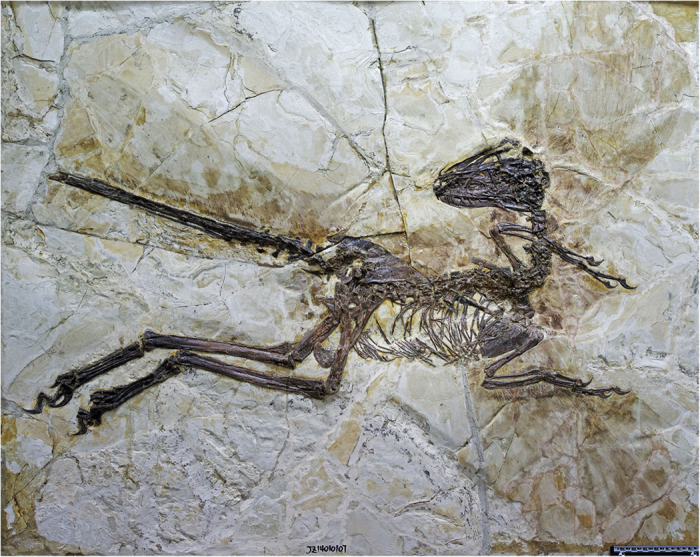
The holotype of the large-bodied, short-armed Liaoning dromaeosaurid *Zhenyuanlong suni* gen et. sp. nov. (JPM-0008).

The type specimen of *Zhenyuanlong* is a large animal for a Liaoning dromaeosaurid, measuring 126.6 cm in total body length as preserved (Fig. 1). However, based on comparison with *Tianyuraptor*^15^, approximately half of the tail is considered missing, meaning that the individual would have been closer to 165 cm in total body length in life. Femur length is a convenient proxy for comparing the size of Liaoning dromaeosaurids, as all Liaoning taxa are known from at least one femur and femur length is a strong correlate with body mass^22^. The femur of *Zhenyuanlong* is 193.4 mm long, compared with 212.5 mm in *Tianyulong*^15^, 153 mm in *Changyuraptor*^10^, 109.8–148 mm in *Sinornithosaurus*^13^,^23^, 107.7 mm in *Graciliraptor*^11^, and 72–97 mm in *Microraptor*^5^,^24^. Therefore, *Zhenyuanlong* is larger than all Liaoning dromaeosaurids except for *Tianyuraptor*, and is considerably larger than most Liaoning dromaeosaurid specimens.

**Figure 2:**
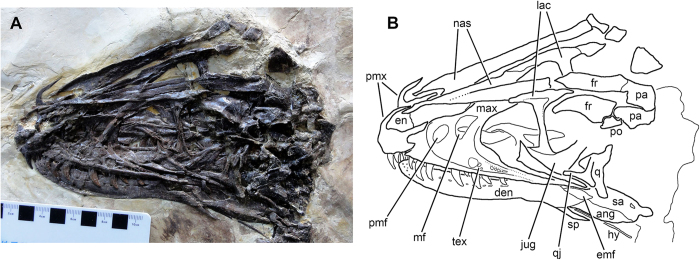
The skull of the large-bodied, short-armed Liaoning dromaeosaurid *Zhenyuanlong suni* gen et. sp. nov. (JPM-0008). (**A**) photograph and (**B**) line drawing. Abbreviations: ang, angular; den, dentary; emf, external mandibular fenestra; en, external naris; fr, frontal; hy, hyoid; jug, jugal; lac, lacrimal; max, maxilla; mf, maxillary fenestra; nas, nasal; pa, parietal; pmf, promaxillary fenestra; pmx, premaxilla; po, postorbital; q, quadrate; qj, quadratojugal; sa, surangular; sp, splenial; tex, pitted texture on lateral surface of antorbital fossa.

The skull of *Zhenyuanlong* is generally well preserved, although the bones of the back portion of the cranium surrounding the lateral temporal fenestra and comprising the braincase are heavily crushed (Fig. 2). On the maxilla, the antorbital fossa is bordered ventrally by a sharp rim, as in *Sinornithosaurus*^21^ but differing from *Tianyuraptor* and most other dromaeosaurids. The lateral surface of the fossa underneath the antorbital fenestra is marked by a series of pits, as is characteristic for Liaoning dromaeosaurids^25^. However, the degree of sculpturing is weak, similar to *Tianyuraptor* and distinct from *Sinornithosaurus*, in which the pits are deeper, more numerous, and extend across almost the entire maxillary antorbital fossa^21^. The promaxillary fenestra is huge, almost equal in size to the maxillary fenestra, and located dorsally at approximately the same level as the maxillary fenestra (Fig. 2). A large promaxillary fenestra is also seen in other Liaoning dromaeosaurids such as *Sinornithosaurus*^21^ and *Tianyuraptor*^15^, but it is much smaller in most other dromaeosaurids, such as *Velociraptor*^26^ and *Tsaagan*^27^. Like *Sinornithosaurus*, but unusual for dromaeosaurids, the maxillary fenestra is not recessed into its own fossa^9^. The lateral lamina of the ascending ramus of the maxilla is extremely reduced to a small triangular exposure in lateral view, as in *Sinornithosaurus* and *Microraptor*, but unlike the much broader lamina in most other dromaeosaurids^9^.

The T-shaped lacrimal appears to have a pneumatic excavation on its lateral surface, where the anterior ramus and ventral ramus merge. The quadratojugal has a pronounced posterior process and a thin dorsal ramus. A pronounced lateral crest on the nasals and frontals forms the lateral edge of the skull roof. The frontals are elongate and the supratemporal fossa extends far anterior onto the dorsal surface of the bone. The parietals are broken but the preserved portions indicate that the left and right elements were fused in life, an unusual feature for dromaeosaurids that is also seen in *Tianyuraptor* and *Sinornithosaurus*^9^. There is a series of large foramina extending across the middle of the lateral surface of the dentary, but these are not set into a distinct groove. The external mandibular fenestra is large and is bordered by the dentary, angular, and surangular. There is a small posterior surangular foramen immediately anteroventral to the glenoid. The splenial is widely exposed in lateral view, and ventral to the posterior end of the lower jaw a long, thin hyoid is preserved.

The neck is complete, and cervical neural arches are X-shaped in dorsal view, with a small centrally placed sheet-like neural spine and widely divergent zygapophyses. The dorsal vertebrae have anteroposteriorly elongate rectangular neural spines, which lack a swelling (‘spine table’) on their dorsal margins. This is also the case in *Tianyuraptor* and *Microraptor*, whereas many other dromaeosaurids have a swelling dorsally^9^. There are six vertebrae in the sacrum, four of which are clearly fused. A complex network of interwoven bony rods reinforces the tail, as is characteristic of dromaeosaurids^8^, and extends proximally nearly to the base of the tail. The middle caudal vertebrae are approximately three times as long as the dorsal vertebrae, similar to most other Liaoning dromaeosaurids but proportionally longer than in most other dromaeosaurids, in which these caudals are roughly twice as long as the dorsals^9^.

**Figure 3:**
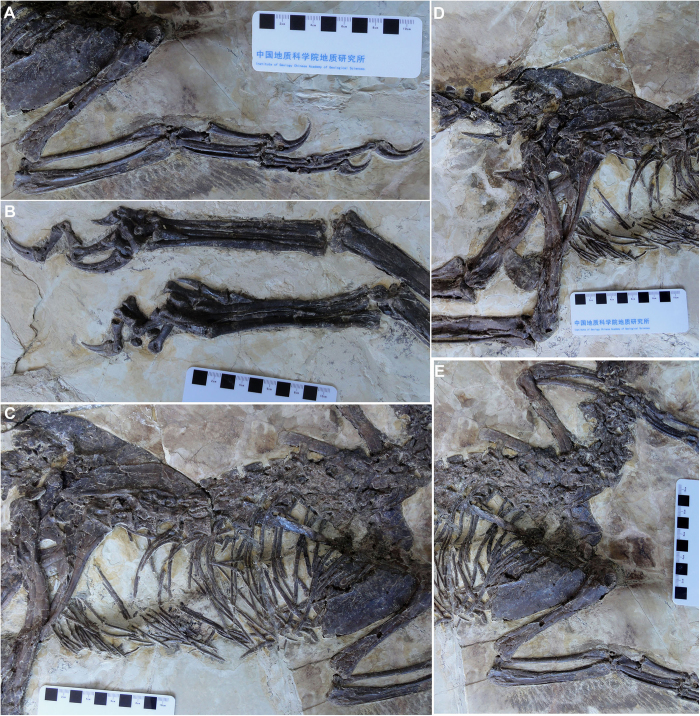
The postcranium of the large-bodied, short-armed Liaoning dromaeosaurid *Zhenyuanlong suni* gen et. sp. nov. (JPM-0008). (**A**) right forearm; (**B**) distal hindlimbs; (**C**) thorax; (**D**) pelvis; (**E**) pectoral girdles.

Both pectoral girdles and forelimbs are preserved in articulation (Fig. 3A,E). Ossified sternal plates are present, but the left and right elements are not fused to each other. The scapular blade is long and strap-like, does not expand distally, and is straight as in *Sinornithosaurus*, not dorsoventrally curved as in most other dromaeosaurids^9^. Little can be said about the coracoid because of poor preservation. The most notable feature of the *Zhenyuanlong* forearm is that it is extremely short. The ratio of the entire forelimb (humerus, ulna, digit II) to the hindlimb (femur, tibia, digit III) is 0.48, which is the shortest of any Liaoning dromaeosaurid. In comparison, this ratio measures 0.53 in the short-armed *Tianyuraptor* and more than 0.80 in every other Liaoning dromaeosaurid specimen^10^,^15^. Similarly, the humerus of *Zhenyuanlong* is the shortest relative to the femur of not only any Liaoning dromaeosaurid, but any dromaeosaurid in general except for the very basal taxon *Mahakala*^28^ and the aberrant unenlagiine *Austroraptor*^29^. The humerus-to-femur ratio of *Zhenyuanlong* is 0.626, compared to 0.65 in *Tianyuraptor* and a ratio greater than 0.75 in all other Liaoning dromaeosaurids^10^,^24^.

The humerus bears a short deltopectoral crest, but other anatomical details are obscured by crushing. The ulna is robust and curved, with a small but marked olecranon process. One of the most distinctive features of *Zhenyuanlong* is its extremely slender radius, which is much thinner than the ulna and also thinner at mid-shaft than the shaft of manual phalanx I-1. The latter is a highly unusual feature among theropods, as such a thin radius relative to the first digit is not seen in any other dromaeosaurids and is only present in alvarezsauroids and a handful of basal coelurosaurs^9^. *Zhenyuanlong* is also unique among Liaoning dromaeosaurids in that the second metacarpal (55 mm) is shorter than the combined lengths of metacarpal I plus the first phalanx of digit I (60 mm).

The pelvic girdles and hindlimbs are essentially complete (Fig. 3B–D). The ilium is anteroposteriorly long and dorsoventrally shallow, with a preacetabular process that is slightly longer than the postacetabular process. The anterior end of the preacetabular process and the posterior edge of the postacetabular process are both pointed, and there is no lobate brevis shelf that projects posteriorly beyond the lateral lamina of the postacetabular process as a discrete flange. The acetabulum is small but widely open medially, and it is bordered dorsally by a prominent supracetabular crest and posteriorly by a large antitrochanter on the ischial peduncle of the ilium, both of which would have prevented the femur from splaying far laterally^30^.

The pubic shaft is anteriorly convex as in *Changyuraptor*, *Microraptor*, and *Sinornithosaurus*, not straight as in *Tianyuraptor*^9^. There is no prominent lateral tubercle on the anterior margin of the pubic shaft. This tubercle, which causes the pubis to have a ‘kinked’ appearance, is present in most Liaoning dromaeosaurids and is often held to be a synapomorphy of a Liaoning dromaeosaurid clade^9^,^18^. It is also absent, however, in *Tianyuraptor*^15^. The ischium is much shorter than the pubis and lacks a distally placed process along the posterior edge of the shaft, which is also absent in *Tianyuraptor* but present in *Microraptor* and *Sinornithosaurus*^9^.

The femur (193.4 mm) is shorter than the tibiotarsus (260.3 mm). A similar ratio of tibiotarsus-to-femur length of approximately 1.30 is seen in most other Liaoning dromaeosaurids, except for the large four-winged *Changyuraptor* (1.10), but differs from the proportions of most other dromaeosaurids in which the tibiotarsus is relatively shorter compared to the femur^24^. The metatarsus is elongate, with the three central metatarsals bunched closely together. Large and curved claws cap the three central digits, and digit I is present but reduced in size, attaching to metatarsal II far distally.

**Figure 4:**
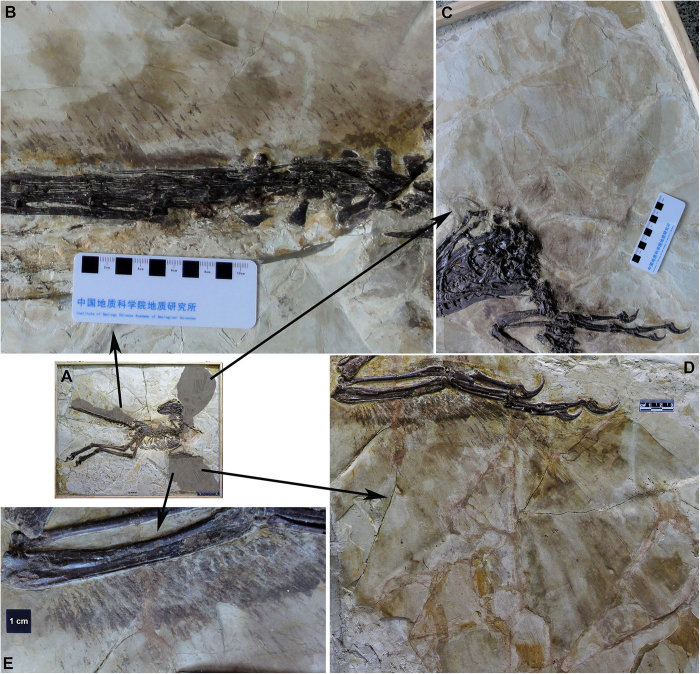
The integument of the large-bodied, short-armed Liaoning dromaeosaurid *Zhenyuanlong suni* gen et. sp. nov. (JPM-0008). (**A**) overview of the skeleton with regions of integument indicated with grey highlight; (**B**) proximal tail; (**C**) left forearm; (**D**) right forearm; (**E**) closeup of coverts on right forearm.

Feathers are present and well preserved on several portions of the body, particularly the arms and tail (Fig. 4). Large pennaceous feathers with a central rachis and barbs form broad wings on the forearms. The shape of the wings is unclear due to taphonomic distortion, but they were evidently quite large, as the preserved portion of the right wing is approximately 800 cm^2^ in area and the left wing is 1120 cm^2^. Fine details of the feathers are better seen on the right wing, where coverts, primaries, and secondaries are visible (Fig. 4C–E). The size, presumed shape, and feather architecture of the wings is generally similar to that of *Microraptor*^5^,^6^, *Changyuraptor*^10^, *Anchiornis*^31^, *Eosinopteryx*^32^, and basal avialans like *Archaeopteryx*^20^,^33^,^34^. The feathered Liaoning dromaeosaurid *Sinornithosaurus*, on the other hand, is known from a specimen that apparently had smaller wings comprised of more simple pennaceous feathers and not a more orderly sequence of coverts, primaries, and secondaries^23^. However, the integument of this specimen is not well preserved and probably does not accurately reflect the wing shape of this species.

On the right forearm wing of *Zhenyuanlong*, a series of approximately 30 small feathers (approximately 25–35 mm long) are preserved attaching to the ulna and metacarpal III. These are the major coverts, the outer layer of feathers that cover the primaries and secondaries dorsally. Most are oriented approximately perpendicular to the ulna, but the most distal ones that connect to metacarpal III are oriented obliquely, such that they approximately parallel the long axis of the hand. It is not clear whether these coverts are complete or broken at their distal ends. However, the fact that nearly all of them are the same length and consistently preserved as broad carbonized impressions, unlike the more posterior wing feathers that are represented almost entirely by faint impressions of the rhachides, suggests that their size and shape are genuine. If so, these coverts are short as in modern birds, not elongate and covering much of the wing as has been hypothesized for the basal avialan *Archaeopteryx* and the close avialan outgroup *Anchiornis*^30^ (but see Foth *et al.*^20^ for an alternative view).

Pennaceous primary and secondary remiges are also identifiable, but are not as well preserved as the coverts. These feathers evidently comprised most of the surface area of the wing, and extend far posterior to the coverts. Counting these feathers is difficult, but there appears to be approximately 10 primaries and 20 secondaries. It is also difficult to measure the lengths of these feathers, both because of poor preservation and because both wings are broken posteriorly. It is clear, however, that both the primaries and secondaries are more than twice the length of the humerus, as in *Microraptor*^5^,^6^ and modern birds, but unlike the shorter primaries and secondaries of the close avialan outgroups *Anchiornis* and *Eosinopteryx*, which are approximately 1.5 times the length of the humerus^31^,^32^. On the better preserved right wing of *Zhenyuanlong* the primaries are longer than the secondaries. The secondaries are oriented approximately perpendicular to the ulna whereas the primaries are positioned at an acute angle to the manus, which becomes less acute and approaches 90 degrees as the feathers progress proximally up the arm. A very similar arrangement is also seen in *Microraptor*^5^. Some primaries and secondaries appear to have an asymmetric shape, but preservation makes this difficult to assess for most of the feathers. There is no sign of any flight feathers attaching to manual digit I to form the alula of modern birds, many Cretaceous birds, and perhaps *Microraptor*^5^.

Large pennaceous feathers are also present on the tail of *Zhenyuanlong* (Fig. 4B). These are preserved only dorsal to the tail. The rhachides are preserved as a series of thin (ca. 1 mm thick), long (up to 8 mm), straight lineations that project approximately 45 degrees posterodorsally from the long axis of the tail. The preservation of the remainder of the feathers is too poor to determine whether they were symmetrical or asymmetrical. The size and orientation of these feathers is similar to the condition in many coelurosaurs bracketing the dinosaur-bird transition, including *Sinornithosaurus*^23^, *Jinfengopteryx*^35^, *Anchiornis*^31^, *Eosinopteryx*^32^, and likely *Archaeopteryx*^20^. However, the rhachides in the proximal portion of the tail of *Zhenyuanlong* appear to be longer than those of *Microraptor*^5^,^6^ and *Changyuraptor*^10^, which had relatively small and simple feathers proximally on the tail but much larger and more complex feathers distally, which expanded outwards into a large fan. Because the distal tail is not preserved in *Zhenyuanlong*, the presence or absence of this fan cannot be assessed.

Small regions of feathery integument are also visible above the back and in front of the neck (Fig. 4C). These regions are very poorly preserved but do contain small, simple filaments that are less than one millimeter thick and at most 30 millimeters long. It is uncertain whether these are simple non-shafted feathers or if they had a more complex series of filaments branching laterally from a small central rachis, as has been observed in the body (non-wing, leg, or tail) feathers of *Sinornithosaurus*^21^ and *Changyuraptor*^10^, among other non-avialan dinosaurs^36^.

There is no sign of any feathers on the legs of *Zhenyuanlong*. Given that feathers are preserved across wide portions of the remainder of the specimen, this may indicate a genuine lack of hindlimb feathers in this new taxon. If so, this would immediately distinguish *Zhenyuanlong* from a growing collection of early avialans and close dinosaurian relatives that had extensive, pennaceous, and often asymmetrical feathers on the hindlimbs, including *Microraptor*^5^, *Changyuraptor*^10^, *Pedopenna*^37^, *Anchiornis*^31^, *Eosinopteryx*^32^, and *Xiaotingia*^38^ among non-avialan dinosaurs and *Archaeopteryx*^20^,^34^ and *Sapeornis*^39^ among early avialans. However, it must be noted that the hindlimbs are part of the second major piece of the slab and no feathers are visible on this piece, whereas numerous feathers are seen on pieces one and three. The lack of feathers on the second piece could be an artifact of taphonomy or have been erased during preparation. Therefore, it cannot be totally ruled out that hindlimb feathers were present in life.

### Phylogenetic Analysis

**Figure 5:**
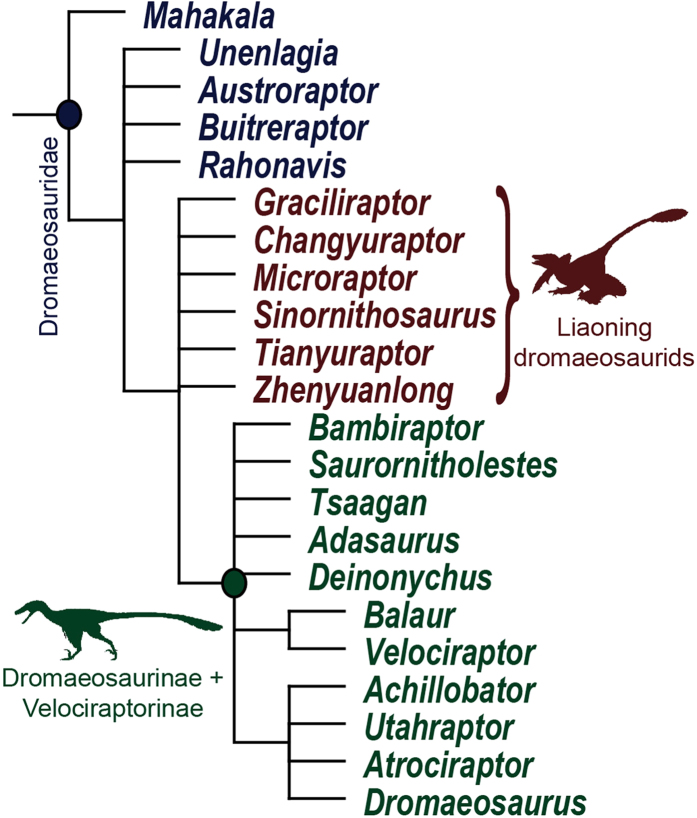
Phylogenetic relationships of *Zhenyuanlong suni* among dromaeosaurid theropods. Strict reduced consensus of dromaeosaurid relationships from 99,999 most parsimonious trees (tree length = 1794, consistency index = 0.285, retention index = 0.726). The entire tree is shown in the Supplementary Information. All dromaeosaurid clades are characterized by Bremer supports of 1 and jackknife supports of less than 50%. Skeletal silhouettes by Scott Hartman and Emily Willoughby, from phylopic.org. [one column]

A phylogenetic analysis recovers the six Liaoning dromaeosaurids (*Changyuraptor*, *Graciliraptor*, *Microraptor*, *Sinornithosaurus*, *Tianyuraptor*, *Zhenyuanlong*) in a basal polytomy with the clade of dromaeosaurines and velociraptorines (Fig. 5). This demonstrates that *Zhenyuanlong* is a dromaeosaurid, and is more closely related to other Liaoning dromaeosaurids and the Laurasian dromaeosaurines and velociraptorines than it is to *Mahakala* and the unenlagiines. However, this topology is considerably less resolved than recovered by earlier versions of this analysis that did not include *Zhenyuanlong*, in which the Liaoning dromaeosaurids are placed in a clade (Microraptorinae) that is sister taxon to Dromaeosaurinae + Velociraptorinae^9^. Enforcing the six Liaoning dromaeosaurids to form a clade results in MPTs one step longer than the shortest trees in the unconstrained analysis. Furthermore, it is noteworthy that *Zhenyuanlong* and *Tianyuraptor* do not form a clade, despite their shared possession of a unique short-armed bauplan.

## Discussion

The discovery of *Zhenyuanlong* increases the already substantial diversity of Liaoning dromaeosaurids, as it is the sixth definitive dromaeosaurid taxon from the famous Jehol Biota. Such high diversity may seem alarming, but the Liaoning dromaeosaurids are found in multiple formations (Yixian and Jiufotang formations) and it is not clear which taxa were directly contemporaneous, so it is possible (and indeed probable) that not all of these species lived together. Regardless, even if all six of these feathered and bird-like taxa did overlap in time and space, this would not be unusual in the context of countless modern ecosystems that support numerous species of birds.

*Zhenyuanlong* is an aberrant and rare animal compared to the vast majority of other Liaoning dromaeosaurids, due to its large body size and proportionally tiny forearms. It is only the second published example of a large, short-armed Liaoning dromaeosaurid, along with the holotype of *Tianyuraptor*^15^. In contrast, other Liaoning dromaeosaurids like *Microraptor* and *Sinornithosaurus* are roughly housecat-sized animals with large forelimbs that are nearly as long as the hindlimbs. These taxa are known from large numbers of specimens found at many sites in Liaoning. Elongate forelimbs are also present in nearly all other dromaeosaurids, and commensurate with the phylogenetic position of dromaeosaurids as close relatives of birds, are considered as something of an intermediate between the short forelimbs of most theropod dinosaurs and the very long arms of living birds which are usually longer than the hindlimb. Both *Tianyuraptor* and *Zhenyuanlong* break the mould for dromaeosaurids and represent an unusual bauplan within the clade.

The discovery of *Zhenyuanlong* complicates the phylogeny of Liaoning dromaeosaurids, and dromaeosaurids as a whole. Perhaps surprisingly, *Zhenyuanlong* and *Tianyuraptor* do not form a clade of short-armed dromaeosaurids in our phylogenetic analysis, nor do all Liaoning dromaeosaurids form their own clade. Instead, there is a large polytomy that includes all Liaoning dromaeosaurids and the clade of Laurasian dromaeosaurines and velociraptorines. In some most parsimonious trees the Liaoning dromaeosaurids form a clade, but in others various species are more closely related to the dromaeosaurine and velociraptorine clade. Similarly, in some most parsimonious trees, *Zhenyuanlong* and *Tianyulong* are sister taxa, but in others they are not. This uncertainty is caused by a large degree of homoplasy, particularly new homoplasy introduced by *Zhenyuanlong*. Although *Zhenyuanlong* and *Tianyulong* are similar to each other in many aspects, *Zhenyuanlong* also shares a hodge-podge of characters with other Liaoning dromaeosaurids exclusive of *Tianyulong*, and some Liaoning dromaeosaurids such as *Zhenyuanlong* share features with velociraptorines and dromaeosaurines that are not present in all Liaoning taxa. This is congruent with the observation that Liaoning dromaeosaurids often differ from each other in subtle ways^25^. As a result, it is not currently certain whether there is a clade of Liaoning dromaeosaurids and a group of short-armed taxa within this group. Liaoning dromaeosaurids may simply be a grade on the line to velociraptorines and dromaeosaurines, and short arms may have evolved multiple times in this grade.

Regardless of the precise phylogenetic relationships of dromaeosaurids, *Zhenyuanlong* provides the first glimpse of feather morphologies in a short-armed dromaeosaurid. Feathers are not preserved on the holotype of *Tianyuraptor*, and the shortness of the forearm in this taxon led to the suggestion that its arms lacked aerodynamic function^15^. Although the arms of *Zhenyuanlong* are short, they supported large and complex wings comprised of pennaceous coverts, primaries, and secondaries, some of which are asymmetric. Whether these wings served any type of aerodynamic function is a separate question that can only be answered with biomechanical analysis, but the wings of *Zhenyuanlong* are strikingly similar to those of *Microraptor* in general size, morphology, and composition, albeit they are supported by much smaller arms.

The integumentary similarities between *Zhenyuanlong* and *Microraptor*-type animals could suggest one of several explanations. First, the large short-armed dromaeosaurids may have had some volant abilities, unrecognized previously because *Tianyuraptor* was preserved without feathers and its small arms were assumed to be un-flightworthy^15^. Perhaps there was not a large functional and behavioural gap between animals like *Microraptor* and *Zhenyuanlong*. We find this unlikely, however, given the striking differences in body size between them, and the incredibly short arms of *Zhenyuanlong* which do not appear optimized for flight (although we reiterate that biomechanical modelling is needed to properly test this). Alternatively, the integumentary similarities between small and clearly volant dromaeosaurids^7^ and larger and presumably non-volant dromaeosaurids could suggest that the larger and short-armed *Zhenyuanlong* evolved from more volant ancestors and maintained a many aspects of the integument through the inertia of common descent or for other selective reasons, not because it needed them for flight. It may be that such large wings comprised of multiple layers of feathers were useful for display purposes^40^, and possibly even evolved for this reason and not for flight, and this is one reason why they may have been retained in paravians that did not fly.

There are over 50 non-avialan paravians currently known from reasonable fossils, and these exhibit a great range of size, osteology, limb proportions, and integumentary covering^41^. The finding of a short-armed dromaeosaurid with large wings comprised of layers of pennaceous feathers adds a previously unrecognized morphology into the mix. Most of these paravians, including all of the Liaoning taxa, lived tens of millions of years after birds split off from other dinosaurs, a long amount of time that would have allowed for diversification in size, proportions, and integument independent of birds. Therefore, we should be cautious in using only one or a few of these feathered paravians as a proxy for the ancestral morphological, functional, and behavioural conditions of birds. *Microraptor* has emerged as a valuable proxy because of the availability of many well-preserved specimens, which have been described in detail^5^,^6^ and studied biomechanically^7^. But it is unclear whether *Microraptor* alone can give much insight into the origin of flight.

*Zhenyuanlong* is a pertinent reminder that not all dromaeosaurids conformed to the same body plan, that larger and short-armed dromaeosaurids likely coexisted with more ‘conventional’ small-bodied and long-armed taxa in the Jehol Biota, that even dinosaurs whose skeletons may not look to be optimized for flight can still possess large and complex wings, that feather types and morphologies were widely variable in species bracketing the dinosaur-bird transition, and that even the best-sampled fossil assemblages can still yield surprising new taxa and specimens with important new anatomical information.

## Methods

### Specimen

The specimen JPM-0008 (Fig. 1) was donated to the Jinzhou Paleontological museum (JPM) in Jinzhou City of Liaoning Province by a local farmer, who is not willing to reveal his identity. After its donation, the specimen was prepared by Y.Q. Zhang at the Institute of Geology, Chinese Academy of Geological Sciences, under the direction of JLü. The holotype of *Zhenyuanlong* is preserved on a slab of fissile siltstone, not as a part and counterpart on two slabs. The slab is comprised of three large pieces: the first includes the head, pectoral girdles and arms, neck, and anterior part of the back and ribcage; the second includes the posterior part of the back and ribcage and the pelvic girdles and legs; and the third includes the proximal-middle portion of the tail. The first and second pieces fit together smoothly, with the bones on each piece articulating with those on the adjoining piece. The fit between the third piece (tail) and the second piece (pelvis) is not as smooth, and some bone may be missing between them. The amount of missing bone is minimal, however, as the third piece includes the posteroventral corner of the left ilium, which very closely articulates with the remainder of the ilium on the second piece. The close fits of the three pieces and the careful preparation done at the Institute of Geology, Chinese Academy of Geological Sciences support the authenticity of the skeleton and plumage.

### Phylogenetic analysis

We included *Zhenyuanlong* in the phylogenetic dataset of Han *et al.*^10^, based on the earlier analysis of Turner *et al.*^9^, which is one of the latest versions of the Theropod Working Group dataset^4^,^41^. This analysis includes 116 taxa (two outgroups, 114 ingroup coelurosaurs) scored for 474 active phenotypic characters. Following Han *et al.*^10^, characters 6, 50, and 52 in the full dataset were excluded, 50 multistates were treated as ordered, and *Unenlagia* was included as a single genus-level OTU.

The analysis was conducted in TNT v1.1^42^ with *Allosaurus* as the outgroup. The dataset was first subjected to a New Technology search (with default parameters for sectorial search, ratchet, tree drift, and tree fusion), which recovered a minimum length tree in 10 replicates. This returned 67 most parsimonious trees (MPTs) of 1794 steps (consistency index = 0.285, retention index = 0.726). These 67 MPTs were then subjected to traditional TBR branch swapping, which resulted in 99,999 total MPTs. This procedure first samples as many tree islands as possible (New Technology search) and then more fully explores each of these tree island (Traditional search). Jackknife resampling (1,000 replicates, 36% character removal probability) and Bremer supports were used to assess clade support. *Hesperonychus*, *Pyroraptor*, and *Shanag* were identified as wildcards and were removed a posteriori in the strict consensus topology.

### Data archiving

Specimen measurements, the phylogenetic character scores for *Zhenyulong*, and the full phylogenetic topologies are available as Supplementary Information.

## Additional Information

**How to cite this article**: Lü, J. and Brusatte, S. L. A large, short-armed, winged dromaeosaurid (Dinosauria: Theropoda) from the Early Cretaceous of China and its implications for feather evolution. *Sci. Rep.* **5**, 11775; doi: 10.1038/srep11775 (2015).

## Supplementary Material

Supplementary Information
