# Supplementary material for: A large, short-armed, winged dromaeosaurid (Dinosauria: Theropoda) from the Early Cretaceous of China and its implications for feather evolution
Source: Sci Rep. 2015 Jul 16;5:11775. doi: 10.1038/srep11775 (PMC4504142; doi:10.1038/srep11775)
Supplement: Supplementary Information [file srep11775-s1.pdf]

Supplementary Information for:

A large, short-armed, winged dromaeosaurid (Dinosauria: Theropoda) from the Early Cretaceous of China and its implications for feather evolution

Junchang Lü[1], Stephen L. Brusatte[2]

[1]Institute of Geology, Chinese Academy of Geological Sciences, Beijing100037, China; Key Lab of Stratigraphy and Paleontology, Ministry of Land and Resources of China, Beijing 100037, China; [2]School of GeoSciences, University of Edinburgh, Grant Institute, James Hutton Road, Edinburgh EH9 3FE, United Kingdom

1. Specimen measurements
2. Phylogenetic analysis

1. Specimen Measurements

The following measurements are all in millimeters.

Skull
Length (anterior margin of premaxilla to posterior end of lower jaw): 166.1
Maximum dorsoventral depth: 58.2
Antorbital fenestra
Anteroposterior length: 44.8
Maximum dorsoventral depth: 38.6
Lower Jaw
Anteroposterior length: 165.2
Best Preserved Cervical Vertebra
Anteroposterior length: 30.2
Dorsoventral depth: 18.3
Best Preserved Dorsal Vertebra
Anteroposterior length: 23.7
Dorsoventral depth: 13.5
Trunk Vertebrae Sequence
Anteroposterior length: 250
Caudal Vertebrae (proximal to distal)
Anteroposterior lengths: 23.6, 23.6, 23.6, 29.7, 32.4, 37.3, 43.4, 40.7, 40.7, 42.1, 46.8, 46.8
Humerus (right)
Proximodistal length: 121.1
Midshaft width: 11.1
Ulna (right)
Proximodistal length: 102.9
Midshaft width: 9.5
Radius (right)
Proximodistal length: 96.6
Midshaft width: 5.0
Metacarpal I(right)
Proximodistal length: 19.5
Midshaft width: 5.1
Metacarpal II (right)
Proximodistal length: 55.7
Midshaft width: 5.4
Metacarpal III (right)
Proximodistal length: 53.0
Midshaft width: 2.7
Phalanges of First Manual Digit (right)
Proximodistal lengths: 40.5, 25.4
Midshaft widths: 4.4, -
Phalanges of Second Manual Digit (right)

Proximodistal lengths: 32.8, 39.2, 31.7
Midshaft widths: 5.2, 4.5, -
Phalanges of Third Manual Digit (right)
Proximodistal lengths: 14.9, 8.1, 24.7, 22.8
Midshaft widths: 2.8, 3.2, 3.2, -
Ilium
Anteroposterior length: 128.3
Maximum dorsoventral depth: 29.3
Pubis
Proximodistal length: 155.7
Ischium
Proximodistal length: 80.1
Femur (right)
Proximodistal length: 193.4
Midshaft width: 19.0
Tibia (right)
Proximodistal length: 260.3
Midshaft width: 17.9
Fibula (right)
Proximodistal length: 262.5
Midshaft width: 3.1
Metatarsals I-IV (right)
Proximodistal lengths: 27.7, 128.3, 129.8, 128.2

2. Phylogenetic Analysis

We included *Zhenyuanlong* in the phylogenetic analysis of Han et al. (2014) (see Methods). The following are the scores for *Zhenyuanlong*.

10?????????????????10000??1110?0??10001?110011??????????????????00??0100?11??0?0100
001010000???0?11???????00?1????01?0?1????01?01?????0???1??0???1010000110221???1110
11?3010111?21120??????0??0???????00??1?10?00100?00????0?0???00??00011?0100000111
?00?1000??00000??1?10000?000???????????????????????0????0?0????0?0?0?????0?0??00????
10???????????????????00??00????0?0?????????????????????010?10?0???00000?00?0??0?000?
?????0????00?000?0?0???01??110000?????????11?110?0????????????0

The only other changes to the Han et al. (2014) analysis are as follows:

-Changed *Tianyuraptor* from 1 to 0 for character 231
-Changed *Tianyuraptor* from 1 to 0 for character 412
-Changed *Microraptor* from 0 to 1 for character 1
-Changed *Changyuraptor* from 1 to 0 for character 236
-Changed *Changyuraptor* from 2 to 1 for character 266

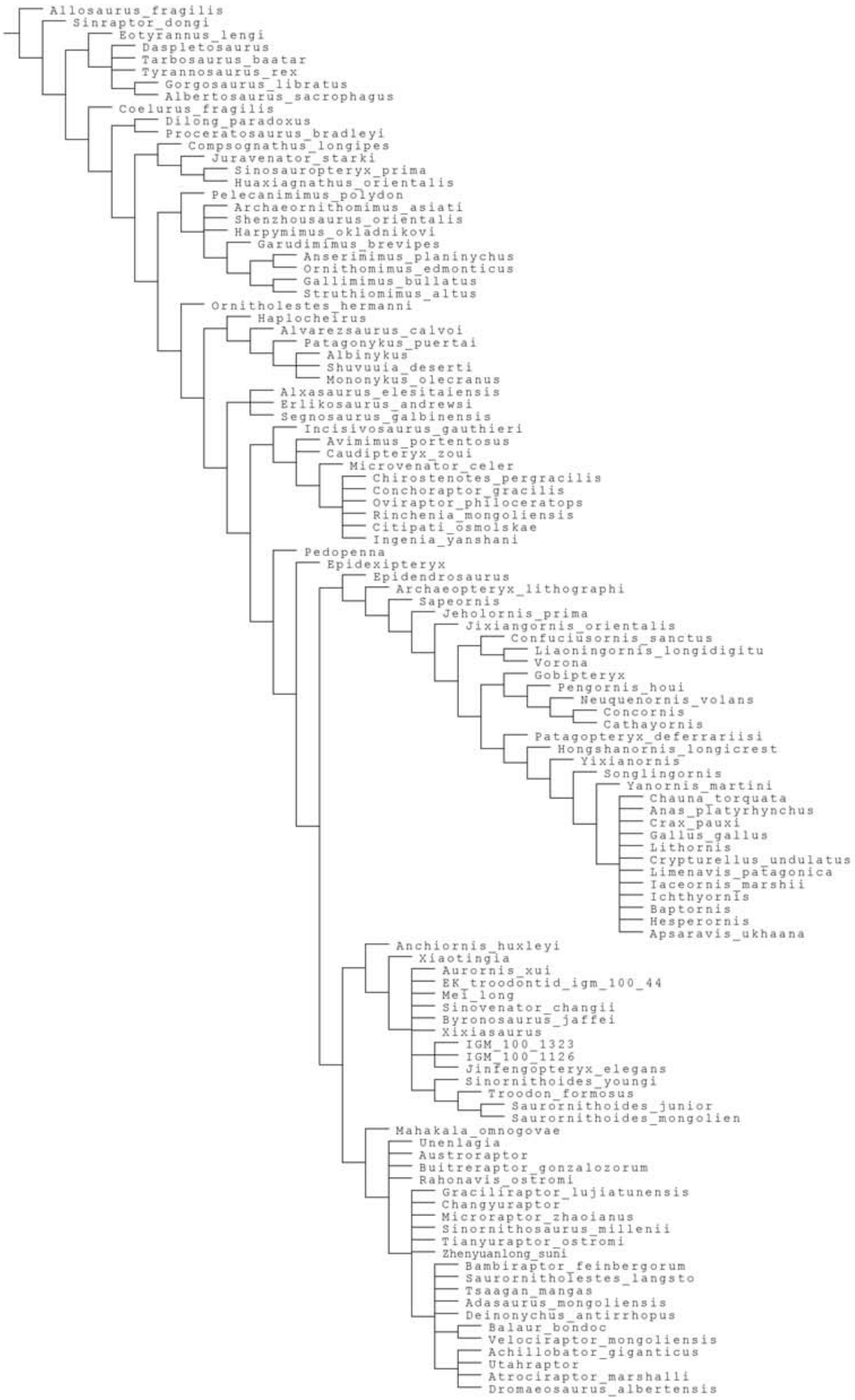
Allosaurus_fragilis
Sinraptor_dongi
Eotyrannus_lengi
Daspletosaurus
Tarbosaurus_baatar
Tyrannosaurus_rex
Gorgosaurus_libratus
Albertosaurus_sacrophagus
Coelurus_fragilis
Dilong_paradoxus
Proceratosaurus_bradleyi
Compsognathus_longipes
Juravenator_starki
Sinosauropteryx_prima
Huaxiagnathus_orientalis
Pelecanimimus_polydon
Archaeornithomimus_asiati
Shenzhousaurus_orientalis
Harpymimus_okladnikovi
Garudimimus_brevipes
Anserimimus_planinychus
Ornithomimus_edmonticus
Gallimimus_bullatus
Struthiomimus_altus
Ornitholestes_hermanni
Haplocheirus
Alvarezsaurus_calvoi
Patagonykus_puertai
Albinykus
Shuvuuia_deserti
Mononykus_olecranus
Alxasaurus_elesitaiensis
Erlikosaurus_andrewsi
Segnosaurus_galbinensis
Incisivosaurus_gauthieri
Avimimus_portentosus
Caudipteryx_zoui
Microvenator_celer
Chirostenotes_pergracilis
Conchoraptor_gracilis
Oviraptor_philoceratops
Rinchenia_mongoliensis
Citipati_osmolskae
Ingenia_yanshani
Pedopenna
Epidexipteryx
Epidendrosaurus
Archaeopteryx_lithographi
Sapeornis
Jeholornis_prima
Jixiangornis_orientalis
Confuciusornis_sanctus
Liaoningornis_longidigitu
Vorona
Gobipteryx
Pengornis_houi
Neuquenornis_volans
Concornis
Cathayornis
Patagopteryx_deferrariisi
Hongshanornis_longicrest
Yixianornis
Songlingornis
Yanornis_martini
Chauna_torquata
Anas_platyrhynchus
Crax_pauxi
Gallus_gallus
Lithornis
Crypturellus_undulatus
Limenavis_patagonica
Iaceornis_marshii
Ichthyornis
Baptornis
Hesperornis
Apsaravis_ukhaana
Anchiornis_huxleyi
Xiaotingia
Aurornis_xui
EK_troodontid_igm_100_44
Mei_long
Sinovenator_changii
Byronosaurus_jaffei
Xixiasaurus
IGM_100_1323
IGM_100_1126
Jinfengopteryx_elegans
Sinornithoides_youngi
Troodon_formosus
Saurornithoides_junior
Saurornithoides_mongolien
Mahakala_omnogovae
Unenlagia
Austroraptor
Buitreraptor_gonzalozorum
Rahonavis_ostromi
Graciliraptor_lujiatunensis
Changyuraptor
Microraptor_zhaoianus
Sinornithosaurus_millenii
Tianyuraptor_ostromi
Zhenyuanlong_suni
Bambiraptor_feinbergorum
Saurornitholestes_langsto
Tsaagan_mangas
Adasaurus_mongoliensis
Deinonychus_antirrhopus
Balaur_bondoc
Velociraptor_mongoliensis
Achillobator_giganticus
Utahraptor
Atrociraptor_marshalli
Dromaeosaurus_albertensis

Figure S1 | Phylogenetic relationships of *Zhenyuanlong suni* among coelurosaurian theropods. Strict reduced consensus from 99,999 most parsimonious trees (tree length=1794, consistency index=0.285, retention index=0.726).
